# Supplementary material for: Reconciling Mining with the Conservation of Cave Biodiversity: A Quantitative Baseline to Help Establish Conservation Priorities
Source: PLoS One. 2016 Dec 20;11(12):e0168348. doi: 10.1371/journal.pone.0168348 (PMC5173368; doi:10.1371/journal.pone.0168348)
Supplement: S1 Dataset — (ZIP) [file pone.0168348.s002.zip › Taxa/Serra Norte/SN_2013/N5S/Lista N5S-36.pdf]

CAVIDADE N5S-0036

Classe	Ordem	Fam/Outros	Gên/Outros	Espécie	S-U	U-S	Zon.
Annelida	Oligochaeta			jovem	1		E
Arachnida	Acari	Ixodidae		jovem	X		E
Arachnida	Araneae	Araneidae		jovem	X	X	E
Arachnida	Araneae	Corinnidae		jovem	1		E
Arachnida	Araneae	Ctenidae	*Ctenus*	sp.3	1		E
Arachnida	Araneae	Drymusidae		jovem	X		E
Arachnida	Araneae	Mysmenidae	*Microdipoena*	sp.1	X		E
Arachnida	Araneae	Pholcidae		jovem		X	E
Arachnida	Araneae	Salticidae		jovem		X	E
Arachnida	Araneae	Theridiidae		jovem	X		E
Arachnida	Araneae	Theridiosomatidae	*Plato*	sp.1		X	E
Entognatha	Collembola	Paronellidae		sp.1	X		E
Entognatha	Collembola	Paronellidae		sp.2		X	E
Insecta	Coleoptera	Dytiscidae		sp.6		X	E
Insecta	Coleoptera	Dytiscidae		sp.7		X	E
Insecta	Coleoptera			jovem	X		E
Insecta	Diptera	Chironomidae		sp.	X		E
Insecta	Diptera	Chloropidae		sp.	X		E
Insecta	Diptera	Psychodidae	Phlebotominae	sp.	X		E
Insecta	Hymenoptera	Formicidae	*Camponotus*	sp.1	X		E
Insecta	Hymenoptera	Formicidae	*Camponotus*	sp.2	X	X	E
Insecta	Hymenoptera	Formicidae	*Dolichoderus*	aff. *bispinosus*		X	E
Insecta	Hymenoptera	Formicidae	*Pachycondyla*	sp.2		X	E
Insecta	Hymenoptera	Formicidae	*Paratrechina*	sp.1		X	E
Insecta	Hymenoptera	Formicidae	*Pheidole*	sp.2	X		E
Insecta	Hymenoptera	Formicidae	*Solenopsis*	sp.1	X	X	E
Insecta	Orthoptera	Phalangopsidae	*Paraclodes*	sp.	2	1	E
Amphibia	Anura			sp.		X	
Mammalia	Chiroptera	Emballonuridae	*Peropteryx*	*kappleri*	1	1	E
Reptilia	Squamata	Gekkonidae	*Thecadactylus*	sp.	1		E
